# Book Notices

**Published:** 1893-01

**Authors:** 


### Book Notices.

INTERNATIONAL CLINICS: A Quarterly of Clinical Lectures on Medicine, Neurology, Pediatrics, Surgery, Genito-Urinary Surgery, Gynæcology, Ophthalmology, Laryngology, Otology and Dermatology, by Professors and Lecturers in the Leading Medical Colleges of the United States, Great Britain and Canada. Edited by Jno. M. Keating, M. D., Colorado Springs, Col., Fellow of College of Physicians, Philadelphia, etc., etc.;

Judson Deland, M. D., Philadelphia, Instructor in Clinical Medicine and Lecturer on Physical Diagnosis and Symptomatology, University Pennsylvania, etc.; J. Mitchell Bruce, M. D., F. R. C. P., London, Physician and Lecturer on Therapeutics at Charing Cross Hospital, etc.; David W. Finlay, M. D., F. R. C. P., Aberdeen, Scotland, Professor of Practice of Medicine, University of Aberdeen, etc. J. P. Lippincott & Co., Philadelphia, 1892.

Volumes 1, 2 and 3 of second series, 1892, are received. These lectures are culled, with discriminating care, from the best clinical sources in America, Great Britain and Canada, and are arranged so as to make a pretty full and complete treatise on each subject discussed; and collectively, they cover the entire field in each department of medicine and surgery. They have the great advantage over even the latest and best and most carefully reviewed text-books, in that they reflect the most advanced views, theories and practices of the real working men—the progressive, thinking men in each department. These men, by their labor, research and lectures, together with their written contributions to current literature have become distinguished, and are all recognized as authority. In the presentation of these lectures fresh from the clinics of the great medical centres of English speaking people, Messrs. Lippincott have done a great work; one which should earn for them the gratitude of the American profession. It is like serving hot cakes while they are hot. If a man cannot go to Philadelphia, New York or London to take a post-graduate course, he can have the post-graduate course right at home by his own fireside, in his own office. He can, for $2.75, get a handsome volume, substantially bound, containing the lectures of Pepper, DaCosta, Roosa, Agnew, Ashhurst, of America, Charcot or Pean, Mackenzie or Macdonald or any of the great teachers, carefully edited and revised, and can "read, mark and inwardly digest" them at leisure; and, moreover, by taking the whole series he can read up to date on any and all subjects.

It would be tedious, as it is unnecessary, to give a summary of the contents of each of these volumes. We have said enough to give an idea of their contents; and we can only add that it is almost equal to attending an extra course, to read this valuable work. Volume 2 contains a portrait of the lamented *Agnew*, and a biographical sketch by Dr. John Ashhurst. Write to J. B. Lippincott & Co. for the set, if their handsome little agent fails to call on you.

---

AN AMERICAN TEXT-BOOK OF THE MEDICAL AND SURGICAL DISEASES OF CHILDREN. By Drs. J. M. DaCosta, Wm. Pepper, Philadelphia; S. S. Adams, Washington; John Ashhurst, Jr., Philadelphia; A. D. Blackador, Montreal, Can., and sixty other eminent clinicians. In preparation. For sale by subscription only. W. B. Saunders, Publisher.

The JOURNAL will announce its advent soon. This will be a popular work.

---

THE AMERICAN TEXT-BOOK OF SURGERY, edited by Professors Keen and White, of Philadelphia, which has only been issued a few months, is already a phenomenal success. It has been adopted as a text-book by forty-nine of our leading medical colleges and universities. Nearly five thousand copies have been placed in physician's libraries, and every indication points to a sale of at least as many copies more in the next six months.

Dr. Nicholas Senn, of Chicaco, is now preparing a "Syllabus of Lectures on the Practice of Surgery," arranged in conformity with the "American Text-Book of Surgery," which will be a valuable aid to all who have this great book.

